# Effect of self-management of stroke patients on rehabilitation based on patient-reported outcome

**DOI:** 10.3389/fnins.2022.929646

**Published:** 2022-10-28

**Authors:** Yongsheng Sun, Chengjiang Liu, Nianping Zhang, Debing Yang, Jun Ma, Cungen Ma, Xi Zhang

**Affiliations:** ^1^The Second Medical Center of the General Hospital of the People’s Liberation Army, Beijing, China; ^2^Medical College of Shanxi Datong University, Datong, China; ^3^Department of General Medicine, Affiliated Anqing First People’s Hospital of Anhui Medical University, Anhui, China; ^4^College of Educational Science and Technology of Shanxi Datong University, Datong, China; ^5^Research Center of Neurobiology, Shanxi University of Chinese Medicine, Taiyuan, China

**Keywords:** stroke, self-management, PRO, rehabilitation effect, patient-reported

## Abstract

**Objective:**

This study aimed to investigate the effect of self-management behavior on the rehabilitation of stroke patients to lay a theoretical basis for using patient-reported outcome (PRO) for rehabilitation evaluation of stroke patients.

**Materials and methods:**

396 patients hospitalized in the Department of Neurology of 4 tertiary general hospitals in Datong from August 1st 2018 to March 31st 2020 were included in accordance with the inclusion and exclusion criteria. The included patients were randomly assigned into a self-management intervention group and a control group. Only the control group received the clinical pathway intervention of stroke rehabilitation. The stroke patients in the intervention group received the self-management intervention in addition to the clinical pathway intervention of stroke rehabilitation. The self-management status and rehabilitation results of the patients were evaluated in 24 h and 3 months after the patients were enrolled, respectively. Statistical description and analysis were conducted using SPSS20.0 statistical software. The general data of the patients were expressed by percentage. The data regarding patients’ self-management and rehabilitation results were statistically described by percentage, mean and standard deviation. The comparison between groups was drawn through *t*-test and analysis of variance. Bonferroni method was used for multiple comparison correction. The correlation between rehabilitation results and patients’ basic conditions and self-management was investigated through Pearson correlation analysis. The main factors for self-management behaviors were studied through multiple stepwise regression analyses.

**Results:**

The total scores of self-management behaviors of the investigated subjects achieved statistical significance in different ages, occupations, educational levels, income levels, exercise intervention, past medical history, BMI, as well as marital status (*P* < 0.01). In this study, there was no statistical difference in different genders and medical insurance status (*P* > 0.05). The total scores of self-management behaviors and the scores of the respective dimension were positively correlated with the health education, exercise intervention, functional training, psychological intervention, food intake, living habits, and functional training of stroke patients at the recovery stage. Educational level and marital status were positively correlated with the rehabilitation results of patients. The PRO questionnaire for the stroke scored higher in married patients and highly educated patients, and there were statistically significant differences (*P* < 0.01). Family history was negatively correlated with the rehabilitation results of patients. Exercise intervention and functional training were positively correlated with the rehabilitation results of patients.

**Conclusion:**

Education level, health education, food intake, exercise and rehabilitation training, sleep, and psychological intervention were the main factors for self-management behavior in stroke patients at the recovery stage. Self-management interventions can effectively increase the health education level of stroke patients, strengthen their self-confidence in disease self-management, facilitate the establishment of effective self-management behavior of patients, and improve their quality of life and subjective well-being. Stroke PRO scale can be used to evaluate the clinical intervention effect of self-management on stroke patients in multiple dimensions, especially evaluating the improvement of subjective mental and psychological state of patients, thus revealing the intervention effect of self-management on stroke patients comprehensively.

## Introduction

The rehabilitation of stroke patients at the recovery stage (2 weeks to 6 months after onset) is a vital factor in the prognosis and quality of life of stroke patients (Chen and Bai, 2017; Li et al., 2018; Zeng and Pu, 2019). Existing research has suggested that self-management education (e.g., life management, sports management, treatment compliance management, and psychosocial factor management) can enhance the self-efficacy of stroke patients at the recovery stage. Patients can maintain and promote their health, reduce the effect of diseases, and firmly cope with their diseases (Liu, 2019; Kim et al., 2020; He et al., 2022). The higher the self-management level of patients, the better the treatment compliance and the better the rehabilitation effect will be (Gao et al., 2014a; Dong et al., 2018; Shi et al., 2019). Patient-reported outcome (PRO) refers to the directly acquired information from patients in terms of their health status, functional status, and treatment feelings, which can truly indicate the effect of the disease on patients (Reeves et al., 2018; Xu et al., 2018). In this study, the self-management status of patients at the recovery stage was investigated, the disease effect of patients was evaluated using PRO, and the ways to facilitate the self-management of stroke patients were explored to help stroke patients recover all or part of their ability to take care of themselves as soon as possible, gain their confidence in returning to family and society, and effectively improve their quality of life.

## Objects and methods

### Subjects

This study gained approval from the Medical Ethics Committee of Shanxi Datong University. A total of 396 patients with a stroke at the recovery stage from August 1, 2018 to March 31, 2020 in the Fifth, Third, and Second People’s Hospitals of Datong City, Affiliated with Shanxi Datong University and Tongmei General Hospital of Datong City were included as the subjects for investigation.

The inclusion criteria of subjects are elucidated below: ➀ Patients meeting the diagnostic criteria established at the Fourth National Academic Conference on Cerebrovascular Diseases in 1996 (Wang, 1996) and confirmed with cerebral infarction, cerebral hemorrhage, or subarachnoid hemorrhage through cranial CT or magnetic resonance imaging (MRI); ➁ Patients with clear consciousness and stable vital signs at the recovery stage; ➂ Patients capable of communicating with the investigator by words or writing; ➃ Patients voluntarily cooperating with this study and signing an informed consent form.

The exclusion criteria of subjects included patients with severe illness, the degree of ADL deficiency (the Barthel Index Scale, BI) was BI ≤ 20 and BI = 100,unclear consciousness (The glasgow coma scale ≤ 14) (Li et al., 2021), or cognitive dysfunction (The Montreal cognitive evaluation<26, The mini mental state examination<27) (Jia et al., 2017; Qian et al., 2019).

The general demographic characteristics, lifestyle, health status, treatment and intervention of the subjects were investigated and evaluated by the self-made questionnaire. The PRO scale for the stroke developed by Wei Xiaoyuan of Shanxi Medical University was used to evaluate the rehabilitation results of patients. The PRO scale was investigated using the self-rating scale method, and its reliability, validity, and feasibility were examined, which confirmed its high reliability, validity, fairness, and feasibility (Wei et al., 2015).

### Research methods

The serious and responsible medical staff in the neurology department should be recruited as the investigators for unified training. Subjects with a consistent baseline were included in accordance with the inclusion and exclusion criteria. ➀ Patients were randomly assigned to a control group and a self-management intervention group by computer random number method, with 198 patients in the respective group. The patients’ condition was evaluated within 24 h and 3 months after the patients were enrolled by the self-made general questionnaire and the PRO Scale for the stroke. ➁ The patients in the control group received the clinical pathway intervention of stroke rehabilitation in a standard hospitalization period of 22–28 days. According to the specific type, course and main complications of stroke, appropriate clinical treatment measures were selected according to the Clinical Diagnosis and Treatment Guidelines – Neurology; during the standard hospitalization period of 22–28 days, the patients in the intervention group received self-management education while receiving the clinical pathway intervention of stroke rehabilitation. On the day of admission, the third week of hospitalization and the day of discharge, the nurse in charge of the patient carried out self-management education in the ward (e.g., lifestyle education, health education, exercise intervention, functional training, psychological intervention, and medical monitoring) to improve the patient’s self-efficacy, daily functional activities, and eliminate negative emotions. Methods: The form of mobile phone WeChat official account combined with bedside nursing was used for propaganda and education (Zhang, 2012; Zhang et al., 2020). ➂ The self-made general questionnaire and the PRO scale for the stroke were used to reevaluate the situation of the two groups 3 months after admission.

### Statistical methods

After the questionnaire was collected, the data of study materials were entered into an Excel spreadsheet by two people and two computers, and a database for logical examination and correction was built. The statistical description and analysis were conducted using SPSS20.0 statistical software. The general data of patients were statistically denoted as percentages. The data regarding patients’ self-management and rehabilitation results were statistically described by percentage, mean and standard deviation. The comparison between groups was drawn through *t*-test and analysis of variance. Bonferroni method was used for multiple comparison correction. The correlation between rehabilitation results and patients’ basic conditions and self-management was investigated through Pearson correlation analysis. The main factors for self-management behaviors were studied through multiple stepwise regression analyses. When *P* < 0.05, the difference is statistically significant.

## Results

### Single-factor analysis of self-management behavior in stroke patients at the recovery stage

No data loss in the study, for the 396 subjects included, 176 were male (44.5%), and 220 were female (55.5%), aged from 31 to 97 years, with an average age of 65.8 12.5. The demographic data of patients included (e.g., gender, age, occupation, marital status, educational level, medical insurance, income level, marital status, past medical history, comorbidities, family history, Body Mass Index (BMI) and exercise intervention), which may affect the self-management behavior of stroke patients at the recovery stage, were investigated through independent sample *t*-test or one-way analysis of variance. The results indicated that the total scores of self-management behaviors of patients at the recovery stage of stroke achieved statistical significance in different ages, occupations, educational levels, income levels, exercise intervention, comorbidities, BMI and marital status (*P* < 0.01), whereas they did not achieve any statistical difference in different genders, family histories, stroke type and medical insurance (*P* > 0.05), as listed in Table 1.

**TABLE 1:** Analysis of factors of self-management in stroke patients at the recovery stage ($\bar{\text{x}}\,\text{±s}$).

Project	Group	*n*	Score ($\bar{\text{x}}\,\text{±s}$)	*t*	*P* value
Gender	Male	176	75.21 ± 15.46	–0.087	0.925
	Female	220	81.77 ± 20.18		
Age	Under 45 years old	22	82.25 ± 21.54	38.582	*P* < 0.001
	45∼65	162	78.48 ± 18.41		
	Over 65 years old	212	65.80 ± 26.38		
BMI	Light weight	40	83.21 ± 18.62	10.821	*P* < 0.001
	Health	237	84.35 ± 16.21		
	Fat	119	74.85 ± 16.21		
Educational level	Primary and below	156	74.90 ± 16.21	54.241	*P* < 0.001
	Junior school	133	78.80 ± 18.78		
	Secondary/high school	79	84.32 ± 21.04		
	College and above	28	83.33 ± 16.38		
Occupation	Farmer	168	78.02 ± 18.46	6.201	*P* < 0.001
	Worker	109	80.00 ± 20.60		
	Office worker	16	81.64 ± 21.19		
	Professionals	20	84.43 ± 24.08		
	Administrative staff	20	85.36 ± 19.37		
	Individual operator	6	62.00 ± 9.89		
	Other	57	78.00 ± 11.17		
Marital status	Married	341	83.29 ± 15.56	12.620	*P* < 0.001
	Unmarried/divorced/widowed	55	65.80 ± 24.38		
Exercise intervention	Without	139	74.63 ± 21.39	0.169	*P* < 0.001
	Often	152	84.23 ± 17.81		
	Once in a while	105	78.06 ± 21.39		
Income level	Low	212	76.13 ± 18.69	7.548	*P* < 0.001
	Middle	152	81.96 ± 18.29		
	Tall	32	86.86 ± 19.39		
Past medical history	Without	87	72.93 ± 19.39	7.563	0.001
	History of stroke	234	83.61 ± 18.51		
	TIA	20	72.23 ± 15.07		
	Other	55	81.95 ± 15.98		
Medical insurance	Medical insurance for urban workers	18	65.29 ± 18.04	–0.137	0.062
	Medical insurance for urban residents	180	77.20 ± 17.45		
	New rural cooperative medical system	178	82.00 ± 18.31		
	Medical insurance at patients’ expense and others	20	76.97 ± 17.50		
Family history	Without	194	80.42 ± 18.26	–0.352	0.437
	Hypertension	121	94.60 ± 21.86		
	Diabetes	55	81.50 ± 19.16		
	Heart disease	26	75.90 ± 17.29		
Comorbidities	Without	36	82.00 ± 17.89	6.254	0.001
	Have	360	66.40 ± 8.26		

### Patient-reported outcome questionnaire results in the recovery of stroke patients

The PRO scale for the stroke used in this study included four domains and 10 dimensions, including the physiological domain (e.g., somatic symptoms, cognition, verbal communication, and self-help skills), psychological domain (e.g., anxiety, depression and avoid), social domain (e.g., social contacts and family support) and therapeutic area (satisfaction), which involved a total of 46 items. Stroke PRO scale items were all scored using the Likert five-stage scoring method. All negative scores of the respective dimension were converted and then added with the positive scores to develop the total score. The higher the score, the higher the quality of life will be (Wei et al., 2015). Table 2 presents the theoretical framework of PRO for the stroke. Table 3 lists the final PRO scores of patients at the stroke recovery stage before and after the self-management intervention.

**TABLE 2:** Framework structure of patient-reported outcome scale for stroke.

Field	Dimension	Item
Physical domain (PHD)	Somatic symptom (SOS) Cognition (COG) Verbal communication (VEC) Self-Help skills (SHS)	1-, 2-, 3-, 4-, 5-, 6-, 7- 8, 9-, 10-, 13 11-, 12-, 14, 15 16, 17, 18, 19, 20
Psychological domain (PSD)	Anxiety (ANX) Depressed (DEP) Avoid (AVO)	1-, 2-, 3-, 4-, 5- 6-, 7-, 8-, 9-, 10- 11-, 12-, 13-, 14-
Social domain (SOR)	Social contacts (SOC) Family support (FAS)	1-, 2-, 3- 4, 5, 6, 7
Therapeutic area (THA)	Satisfaction (SAT)	1, 2, 3, 4, 5

“-” represents a negative score for the entry.

**TABLE 3:** Patient-reported outcome questionnaire results of stroke patients in the rehabilitation period ($\bar{\text{x}}\,\text{±s}$).

Field	Dimension	Before self-management intervention		After self-management intervention	
		Self-management intervention group	Control group	Self-management intervention group	Control group
**Physical domain (PHD)**					
	Somatic symptom (SOS)	18.92 ± 6.52	19.21 ± 6.45	17.54 ± 6.13	19.78 ± 6.46
	Cognition (COG)	11.51 ± 2.48	11.65 ± 4.70	16.51 ± 2.28	12.41 ± 4.62
	Verbal communication (VEC)	13.52 ± 3.83	11.67 ± 4.50	18.72 ± 3.63	12.81 ± 4.20
	Self-help skills (SHS)	12.05 ± 7.21	12.21 ± 7.76	12.05 ± 7.21	12.76 ± 7.81
**Psychological domain (PSD)**					
	Anxiety (ANX)	14.03 ± 5.55	13.59 ± 5.10	13.03 ± 4.55	14.37 ± 4.86
	Depressed (DEP)	16.05 ± 4.73	16.06 ± 4.01	16.98 ± 5.73	16.12 ± 3.87
	Avoid (AVO)	13.03 ± 3.93	13.29 ± 3.75	12.03 ± 3.93	13.43 ± 3.81
**Social domain (SOR)**					
	Social contacts (SOC)	13.71 ± 4.04	13.11 ± 3.76	9.16 ± 4.01	8.67 ± 3.60
	Family support (FAS)	17.63 ± 4.36	16.20 ± 3.59	14.71 ± 4.04	13.11 ± 3.76
**Therapeutic area field (THA)**					
	Satisfaction (SAT)	17.63 ± 4.36	16.20 ± 3.59	18.63 ± 4.36	16.21 ± 3.45

**FIGURE 1:**
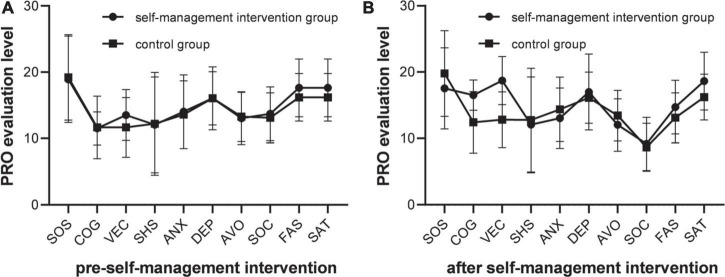
PRO evaluation results comparison. **(A)** The PRO scores was no statistical difference among different groups (*P* > 0.05), the PRO dimension scores of the two group were not statistically significant (all *P* > 0.05). **(B)** The PRO scores was statistical difference among different groups (*P* < 0.05), the PRO dimension scores of the two group were not statistically significant (all *P* > 0.05).

There was no statistical difference in the PRO scores of the self-management intervention group and the control group before the self-management intervention (*P* > 0.05). Statistical difference was found in the PRO scores of the self-management intervention group and the control group after the self-management intervention (*P* < 0.05). After Bonlemmi correction, the PRO scores of the self-management intervention group and the control group after the implementation of self-management intervention were statistically different (*P* < 0.017), and the differences between the other groups were not statistically significant (all *P* > 0.05). Figure 1 presents the comparison of PRO scores of the two groups before and after the self-management intervention.

### Self-management behavior of convalescent stroke patients

Before and after the self-management intervention, the scores of self-management behaviors in 11 dimensions (e.g., food intake, living habits, functional training, monitoring, TCM intervention, health education, exercise intervention, sleep intervention, psychological intervention and oxygen intervention) were compared in the included patients at the rehabilitation stage of stroke (Table 4).

**TABLE 4:** Self-management behavior level and scores of the respective dimension in the self-management intervention group ($\bar{\text{x}}\,\text{±s}$).

Dimension	Before self-management intervention		After self-management intervention	
	Intervention group	Control group	Intervention group	Control group
Food intake	25.65 ± 8.02	24.98 ± 8.60	29.01 ± 9.02	28.78 ± 8.70
Health education	7.33 ± 6.33	7.99 ± 4.33	17.33 ± 6.28	5.02 ± 4.12
Living habits	17.30 ± 6.12	16.71 ± 6.23	17.83 ± 6.30	17.48 ± 6.23
Exercise intervention	10.07 ± 5.68	8.53 ± 4.38	10.92 ± 5.51	4.92 ± 4.38
Monitoring	8.23 ± 5.14	8.93 ± 3.61	8.59 ± 5.68	8.91 ± 3.01
Functional training	9.65 ± 3.85	10.30 ± 5.74	4.91 ± 3.85	11.02 ± 4.26
Sleep intervention	3.61 ± 3.96	3.01 ± 2.28	4.03 ± 3.72	3.11 ± 2.15
Psychological intervention	1.34 ± 2.54	0.91 ± 1.98	1.42 ± 2.05	0.89 ± 2.38
TCM intervention	1.31 ± 1.69	1.21 ± 3.97	1.40 ± 1.52	5.06 ± 3.97
Other	0.85 ± 1.76	0.90 ± 1.84	0.87 ± 1.43	0.86 ± 1.78
Oxygen intervention	0.40 ± 1.16	0.41 ± 1.21	0.40 ± 1.08	0.41 ± 1.15

**FIGURE 2:**
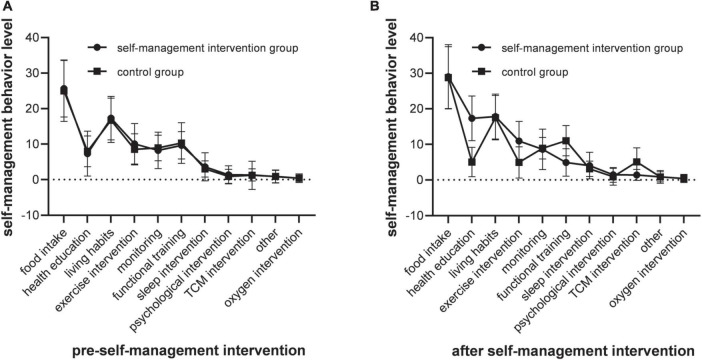
Self-management evaluation results comparison. **(A)** The scores of self-management behaviors were no significant difference among different groups (*P* > 0.05), the dimension scores of self-management intervention were no statistical difference between the other groups (all *P* > 0.05). **(B)** The scores of self-management behaviors were statistically significant differences among different groups (*P* < 0.05), the dimension scores of self-management intervention were no statistical difference between the other groups (all *P* > 0.05).

Before the self-management intervention, there was no significant difference in the scores of self-behavior dimensions among different groups (*P* > 0.05). After the 3-month self-management intervention in the behavioral period, there were statistically significant differences in the scores of self-management behaviors of the two groups (*P* < 0.05). After Bonlemmi correction, there was a statistical difference between the self-management intervention group and the control group in the scores of self-behavior dimensions after the implementation of self-management intervention (*P* < 0.013), and there was no statistical difference between the other groups (all *P* > 0.05). Figure 2 presents the comparison of various behavioral dimensions before and after the self-management between the self-management intervention group and the control group.

### Analysis of factors for self-management behavior pattern of stroke patients in convalescence

The correlation analysis was conducted between the total score of self-management behavior and scores of the respective dimension and a total of 10 dimensions in four fields of the PRO questionnaire. The results indicated that health education, exercise intervention, psychological intervention, food intake, living habits, and functional training of stroke patients at the recovery stage were positively correlated with the total score of self-management behavior and scores of the respective dimension.

Taking the total PRO score of stroke patients as the dependent variable, and introducing the above factors into the equation, a multiple linear regression model was established. The results indicated that there was a relationship between educational level, marital status, family history, exercise intervention, and functional training and the rehabilitation results of stroke patients. Educational level and marital status were positively correlated with the rehabilitation results of patients. Married patients and highly educated patients had high scores in the stroke PRO questionnaire and the differences achieved statistical significance. Family history was negatively correlated with rehabilitation results. The total rehabilitation scores of patients with a family history of stroke were lower than those of patients without a family history, and the differences were statistically significant. Participation in exercise intervention and functional training was positively correlated with the rehabilitation results of patients. Active exercise intervention and functional training can lead to higher scores of rehabilitation results of patients and significant differences. Table 5 lists the results.

**TABLE 5:** Results of multiple linear regression analysis of rehabilitation effects in stroke patients.

Factor	Coefficient of regression	Standard error	*T* value	*P* value	Standardized partial regression coefficient
Educational level	25.745	3.165	8.351	0.000	0.604
Marital status	9.694	4.401	2.203	0.030	0.186
Past medical history	0.938	0.356	2.902	0.004	0.162
Medical insurance	−1.729	1.561	−1.107	0.270	−0.094
Family history	−5.540	3.055	−1.814	0.032	−0.154
Exercise intervention	1.902	0.953	1.996	0.048	0.170
Functional training	8.161	3.034	2.689	0.008	0.227

## Discussion

Stroke recovery patients with a better self-management ability and having mastered several systematic, scientific and effective methods and relevant knowledge and basic rehabilitation skills required for self-management can improve their confidence in self-management of their diseases and play their subjective initiative. They are capable of increasing the rehabilitation results by actively establishing correct self-management behavior patterns (Gao et al., 2014b; He et al., 2017; Huang, 2017).

In this study, there were differences in scores of different dimensions of self-management behavior, and the self-management of food intake was the best, probably because of the traditional concept of “disease entering from the mouth” and the significant treatment effect of diet control for patients. Moreover, the medical staff emphasized the role of the healthy nutrition concept and the significance of long-term adherence during the patients’ hospitalization and discharge education, so the patients’ management of food intake can be effectively performed. The self-management of living habits, exercise intervention, health education, and functional training was better, probably arising from the strengthening of hospital education for patients and popular science education on stroke-related knowledge via the media, newspapers, television and community. Patients developed an awareness that excessive intake of tobacco and alcohol, unreasonable dietary structure, lack of exercise, and emotional abnormalities were significantly correlated with the occurrence and development of stroke. The management time and economic cost in diet, daily life and other aspects were reported to be low and acceptable, thus enabling patients to actively change their dietary structure, conduct the moderate exercise and functional training and maintain a relatively stable and optimistic mood (Li et al., 2016; Yang and Huang, 2016; Hu and Bing, 2019). Special rehabilitation exercise, TCM intervention and disease self-detection were at a poor level, probably due to the limitation of local rehabilitation diagnosis and treatment level and economic income in Datong, thus affecting the dependency on patients’ disease self-monitoring and active rehabilitation exercise. Impacted by China’s medical system and the people’s inherent traditional concept of “emphasizing treatment while neglecting prevention,” the energy and focus are primarily placed on the in-hospital treatment of diseases, while health education on relevant knowledge outside the hospital has been neglected.

Seventy-three percent of the patients surveyed in this study had educational culture below junior middle school level, and their understanding of disease-related knowledge and mastery of rehabilitation training skills were poor, thus resulting in their poor knowledge level of stroke and preventing them from effectively implementing self-management and monitoring of the disease. Accordingly, nursing staff should increase health education for the stroke patients and pay more attention to health education regarding common chronic non-communicable diseases of the elderly and training in rehabilitation skills, so as to help patients understand the significance of self-management after stroke. Furthermore, they should carry out targeted guidance on systematic knowledge and skills in accordance with the patients’ age, educational level, and economic situation, enhance the patients’ behavioral abilities of self-management and scientific rehabilitation training after illness (Yang and Huang, 2016; Hu and Bing, 2019).

The results of multiple regression analysis indicated that marital status of being married, active physical exercise and rehabilitation training were the protective factors for self-management scores of stroke patients; family history, past medical history and complications were the risk factors for self-management of stroke patients. The self-management score of married patients was higher than that of unmarried/divorced/widowed patients. The family relationship and family income of married patients were reported to be relatively stable, and they were significantly supported and encouraged. The patients’ emotions were stable, and they faced stress with a positive attitude, thus improving their self-management ability. The rehabilitation scores of patients actively doing physical exercise were higher than those of patients not doing physical exercise. Regular and appropriate physical exercise improved the physical quality of patients and the rehabilitation of the diseases of stroke patients (Feng et al., 2015; Song et al., 2018; Li et al., 2019). The results indicated that the rehabilitation results scores of patients with no family history were higher than those of patients with a family history of stroke. The disease progression and outcome were affected by genetic and environmental factors. Patients without any family history were not affected by genetic factors and did not have the predisposing factors for the stroke. After active control, the patient’s condition and the rehabilitation results can be easily improved. However, patients with a family history of stroke were relatively difficult to treat and recover (Feng et al., 2015).

This study aimed to investigate the effect of self-management on stroke patients at the recovery stage. Due to the constraints of test time and test conditions, patients from the community and rehabilitation pension institutions were not included, and the sample representation was not comprehensive. When choosing the self-management evaluation scale, no more comprehensive reliability and validity evaluation was conducted due to the test conditions.

## Conclusion

The information was acquired directly from the patients based on the clinical outcomes reported by them, in which the explanation by the medical staff and any other staff was excluded. The information of this study can provide a more valuable reference for doctors in diagnosis and treatment of stroke, and it is of great significance in the clinical treatment practice. For patients at the post-stroke rehabilitation stage, medical intervention in accordance with the feelings and wishes directly from the patients can be the starting point for smooth work.

Education level, health education, food intake, exercise and rehabilitation training, sleep, and psychological intervention were the main factors for self-management behavior in stroke patients at the recovery stage, which revealed that medical staff should improve the health education level of stroke patients and give more support to patients with lower educational levels. Self-management interventions can effectively increase the health education level of stroke patients, help them gain self-confidence in disease self-management, and facilitate the establishment of effective self-management behavior of patients, which is conducive to improving the quality of life and subjective well-being of patients. Stroke PRO scale is a novel sensitive and effective efficacy evaluation tool, which is capable of evaluating the clinical intervention effect of self-management on stroke patients from multiple dimensions, especially evaluating the improvement of the subjective psychological state of patients and thus revealing the intervention effect of self-management on stroke patients comprehensively.

## Limitations

First, this study is only a multicenter study of a regional Grade III hospital, and did not include inpatients in the department of neurology of hospitals below Grade III in the region during the same period; Second, in order to facilitate research, we only used the PRO scale, no other scale is used, such as SF36 scale; Third, the cognitive function of the subjects was only evaluated with MoCA and MMSE neuropsychological evaluation scales, without further hierarchical evaluation. This requires us to further expand the sample in future research. On the basis of operational research, we should try our best to select various evaluation tools to comprehensively evaluate the research object, compare them with other similar research tools, and select the evaluation tool with the best reliability and validity.

## Data availability statement

The raw data supporting the conclusions of this article will be made available by the authors, without undue reservation.

## Ethics statement

The studies involving human participants were reviewed and approved by Medical Ethics Committee of Shanxi Datong University. The patients/participants provided their written informed consent to participate in this study.

## Author contributions

YSS, NPZ, and XZ proposed the idea of the article, formulated the inclusion and exclusion criteria, and wrote the first draft of the manuscript. DBY and JM conducted literature retrieval and screening. YSS and JM conducted data analysis and result writing. CGM and XZ reviewed the first draft of the manuscript. CJL revised the manuscript. All authors contributed to the article and approved the submitted version.
